# Supplementary material for: Allorecognition genes drive reproductive isolation in Podospora anserina
Source: Nat Ecol Evol. 2022 May 12;6(7):910–23. doi: 10.1038/s41559-022-01734-x (PMC9262711; doi:10.1038/s41559-022-01734-x)
Supplement: Supplementary file 1 — Supplementary Methods, Figs. 1–19, Tables 4 and 6 and captions of Tables 1, 2, 3, 5 and 7. [file 41559_2022_1734_MOESM1_ESM.pdf]

nature portfolio

https://doi.org/10.1038/s41559-022-01734-x

Supplementary information

Allorecognition genes drive reproductive isolation in *Podospora anserina*

In the format provided by the authors and unedited

Supplementary Materials for

Allorecognition genes drive reproductive isolation in *Podospora anserina*

S. Lorena Ament-Velásquez, Aaron A. Vogan, Alexandra Granger-Farbos, Eric Bastiaans, Ivain Martinossi-Allibert, Sven J. Saupe, Suzette de Groot, Martin Lascoux, Alfons J. M. Debets, Corinne Clavé, Hanna Johannesson

Correspondence to: lorena.ament@zoologi.su.se and hanna.johannesson@ebc.uu.se

This PDF file includes:

Supplementary Methods
Supplementary Figures S1 to S19
Supplementary Tables 4 and 6
Captions of Supplementary Tables 1, 2, 3, and 5 and 7
References

Other Supplementary Materials for this manuscript include the following:

Supplementary Tables 1, 2, 3, 5 and 7 (.xlsx)

Supplementary Methods

Individual based simulation of the *het-r/het-v* interaction in *P. anserina*

This SLiM v. 3.3.2[1] simulation aims at exploring possible mechanisms through which the interaction of the vegetative incompatibility genes *het-v* and *het-r* can result in the formation of two segregated mating groups within a single population of the fungus *P. anserina*.

Life cycle

The life cycle of *Podospora* is modelled using two populations. This approach is necessary to take into account critical features of the life cycle of *Podospora*. Population 1 represents the diploid stage of the life cycle and population 2 the dikaryotic stage. To complete the life cycle, individuals need to cycle through both populations. Diploid individuals of population 1 transition to population 2 through a selfing phase, which is effectively equivalent to the meiosis leading to the dikaryotic stage in the life cycle of *Podospora*. Individuals of population 2, representing the dikaryotic stage, then produce gametes but are not allowed recombination since in the life cycle of *Podospora* recombination has already occurred at the transition between the diploid and dikaryotic stages. The gametes are then used to form the new diploid individuals of population 1, completing the life cycle.

Genetic architecture

Each individual carries two haploid genomes, including the *het-v* and *het-r* loci on two separate chromosomes. Each locus can be represented by two alleles, *V* and *V1* for *het-v* and *R* and *r* for *het-r*.

Fitness effects of genotypes

The simulation takes into account the main features of the *het-r/v* interaction:

(i) Lethal genotype: We assumed that the *RV* combination within a genotype is lethal. This means that after meiosis dikaryons of the genotype *RV* -XX effectively become monokaryons of genotype XX. This is based on the observation that dikaryons can sometimes disassociate into "sectors" within a culture that have each a different nucleus.

(ii) Balancing selection: Due to vegetative incompatibility, individuals carrying the *R* allele of *het-r* have an advantage over individuals carrying the *r* allele, which is an increasing function of the frequency of *V* in the population (**Supplementary Fig. 16a**). The benefit in relative fitness of *V* over *V1* due to the presence of *R* is identical and also follows the function of panel **Supplementary Fig. 16a** (with frequency of *R* on the x-axis and benefit to *V* on the y-axis). In addition, vegetative incompatibility between *V* and *V1* provides a benefit in relative fitness to the rarer of the two alleles (**Supplementary Fig. 16b**). See SLiM code for fitness functions in the Zenodo repository or in https://github.com/johannessonlab/HetVPaper.

(iii) Sexual incompatibility: fertilization success of two crosses is reduced as a side-effect of the vegetative incompatibility interactions. The interaction between *V* and *R* was set to reduce fertilization success by 40%, which represents a mean of experimentally measured reduction in fertilization success, averaged over parental effects. The interaction of *V* and *V1* also causes a reduction in fertilization success, which we vary from 0% to 40% in the simulations. Due to those

two interactions, fertilization success of a *RV1* x *rV* cross is reduced at least 40% due to *R*/*V* interaction, and up to 80% depending on the cost of the *V*/*V1* interaction.

Structure of a simulation

Each population is composed of 1 000 individuals (2 000 genomes). The selfing rate of dikaryons is fixed for a given simulation. A population is started with uniform genetic background, either *RV1* or *rV*. After 100 generations, 10 individuals (1% of the population) carrying the alternative genotype are introduced. This type of introduction could represent either the migration of individuals from a neighbouring population, or the second stage of an invasion subsequent to *de novo* mutations. We chose not to model explicitly mutation rates as we have no information from the natural system on what such rates could be, and introducing the new genotypes by mutations considerably increases simulation runtime. 300 generations after the introduction of the invading genotype, genotype frequencies are sampled every 20 generations over 100 generations and averaged. We study the effect of the following parameters on the outcome of the simulation (genotype frequencies after invasion): the rate of selfing of the population, the intensity of *V*/*V1* balancing selection, the intensity of *V*/*R* balancing selection and finally the level of prezygotic isolation due to the *V*/*V1* interaction. For each unique parameter combination, 100 replicated simulations are run. The results present the median and distribution of genotype frequencies across the 100 replicated simulations for each parameter combination.

Additional remarks

Fig. 6 and **Supplementary Fig. 17** show the final genotype frequencies after invasion of the *rV* genotype in a population of *RV1* background. **Supplementary Fig. 18** show the reverse invasion scenario. We are primarily interested in the conditions that result in a coexistence of the *rV* and *RV1* genotypes at intermediate frequencies, with the *rV* genotype at low frequency. The *RV* genotype is lethal and therefore absent from the population.

Looking at **Supplementary Figs. 17** and **18**, we can see that the coexistence of the *rV* and *RV1* genotypes is favoured by a high selfing rate. In fact, if selfing rate is low, the invading genotype is unable to enter the population. This is quite intuitive: because the *RV* combination is lethal, an invader carrying the *V* allele in an outcrossing population where all individuals carry *R* has no chance to invade, and vice versa with *R* invading *V*. If selfing rate is high enough however, selective advantage to the rare allele due to vegetative incompatibility kicks in and helps the invasion of the new genotype up to intermediate frequencies. The selfing rate, strength of balancing selection (*V*/*R* and *V*/*V1*) and the strength of prezygotic isolation then all interact to produce the final genotype frequencies after invasion.

We can see that both the *V*/*R* and *V*/*V1* balancing selections favour the invasion of the new genotype and the maintenance of *rV* and *RV1* at intermediate frequencies. The effect of *V*/*V1* balancing selection is particularly visible in **Fig. 6** in the main text, selfing rate 75%. In some cases, invasion is successful but the two dominant genotypes are *rV* and *rV1*. This may occur if the strength of the *V*/*V1* balancing selection is strong enough to overpower the *V*/*R* balancing selection. An increased selfing rate, or increased strength of prezygotic isolation shifts the situation to an *rV* and *RV1* coexistence (see for example **Supplementary Fig. 17a**, selfing 75% and compare the different outcomes with increasing strength of prezygotic isolation).

In conclusion, coexistence of the *rV* and *RV1* genotypes is made possible by high selfing rates and *R*/*V* balancing selection. In addition, *V*/*V1* balancing selection and prezygotic isolation favor that scenario, with prezygotic isolation and selfing contributing to lowered frequencies of the intermediate *rV1* genotype.

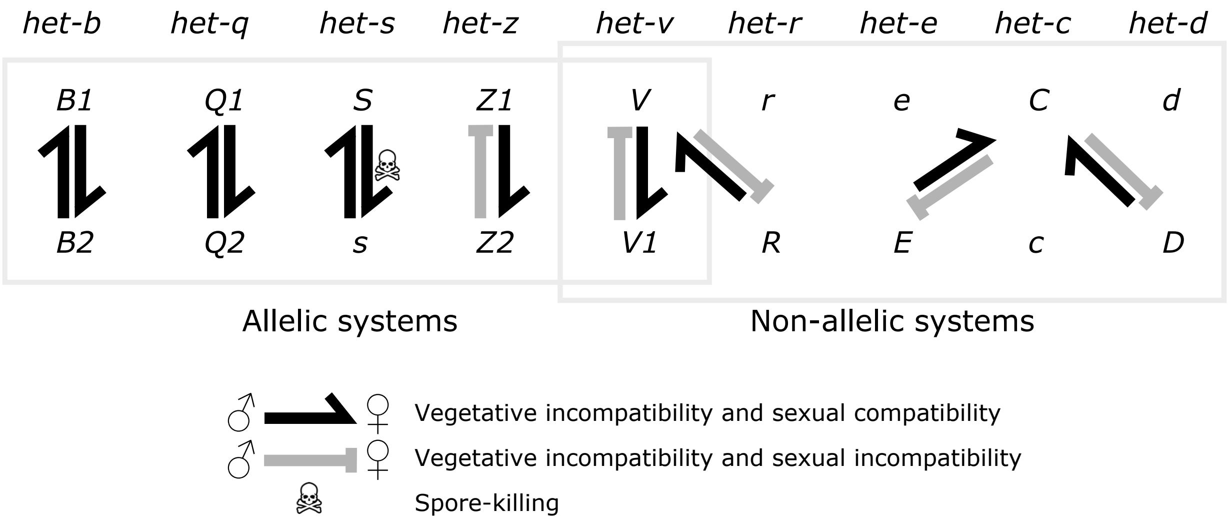

Supplementary Figure 1.

Pleiotropic effects on the sexual function of genetically identified *het* genes in *Podospora anserina*. The diagram gives the two allelic categories (centre) of each of the nine identified *het* loci of *P. anserina* (top). The *het* loci can be classified as allelic or non-allelic systems (*het-v* is involved in two systems). Vegetative and sexual (in)compatibility reactions are given by a black (non-pleiotropic) or grey (pleiotropic) half arrow, whose directions go from the male parent to the female parent. For instance, the diagram specifies that a male *Z2* x female *Z1* cross is sterile while the reciprocal cross is fertile. The skull symbol indicates that a male *S* x female *s* cross can lead to meiotic drive of *s* (killing of *S* spores). For all systems marked as non-allelic, crosses between incompatible genotypes also lead to hybrid lethality, in the form of self-incompatible progeny. Note that *het-c*, *het-d* and *het-e* loci are multi-allelic and that a simplified nomenclature is used here where incompatible interaction are denoted with capital letters, while small letters are non-reactive (neutral) alleles. Based on refs. [2,3].

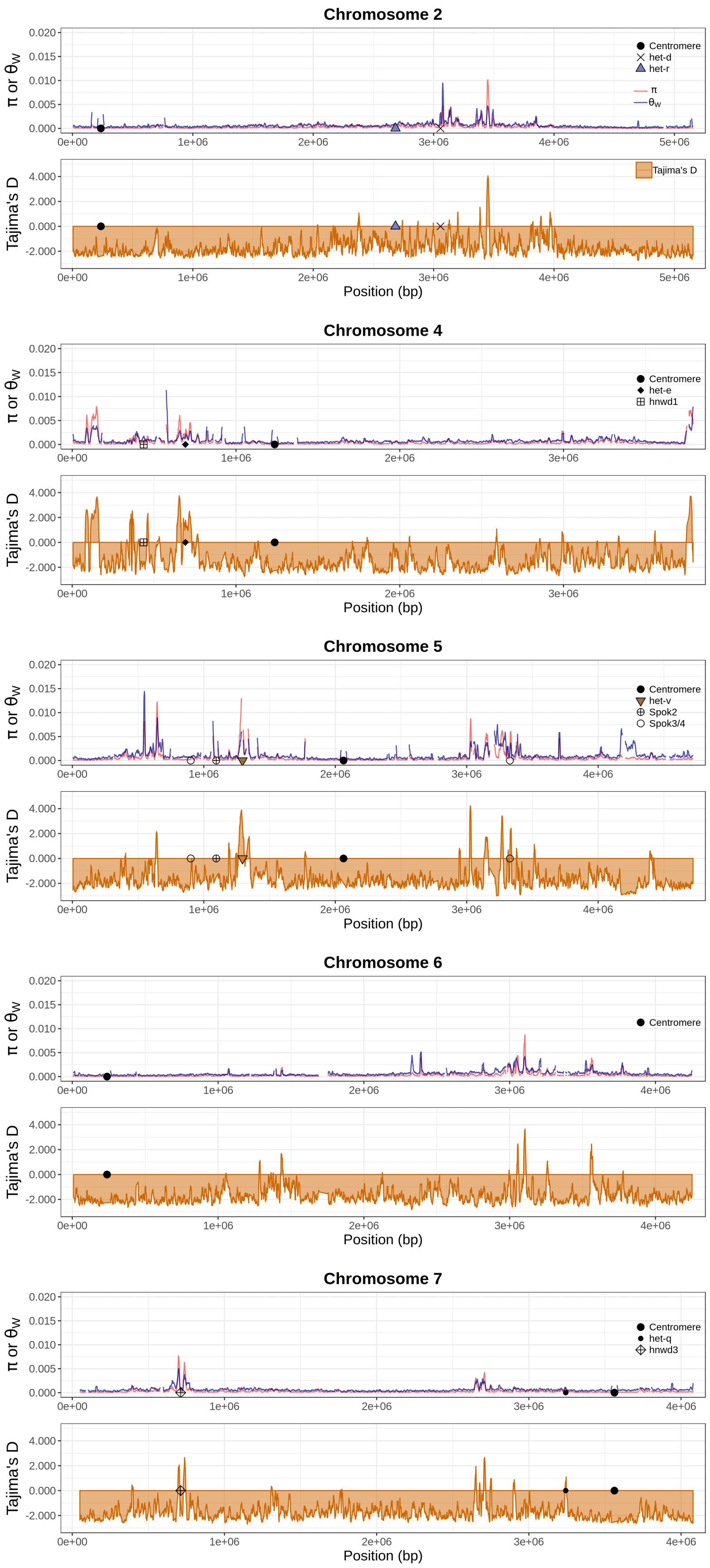
Chromosome 2
π or θW
Tajima's D
Centromere
het-d
het-r
π
θW
Tajima's D
Position (bp)
Chromosome 4
Centromere
het-e
hnwd1
Chromosome 5
Centromere
het-v
Spok2
Spok3/4
Chromosome 6
Centromere
Chromosome 7
Centromere
het-q
hnwd3
0.000
0.005
0.010
0.015
0.020
4.000
2.000
0.000
-2.000
0e+00
1e+06
2e+06
3e+06
4e+06
5e+06

Supplementary Figure 2. (above)

Sliding window analysis (10kb-long with steps of 1kb) of representative chromosomes 2, 4, 5, 6 and 7 with values of genetic diversity (measured as either the pairwise nucleotide diversity π or as Watterson's theta θ_W) and the Tajima's D statistic. Relevant loci are marked, namely *het* genes, the centromere, and meiotic drivers of the *Spok* family. Note that *Spok3* and/or *Spok4* can be found at different locations in the genome depending on the strain.

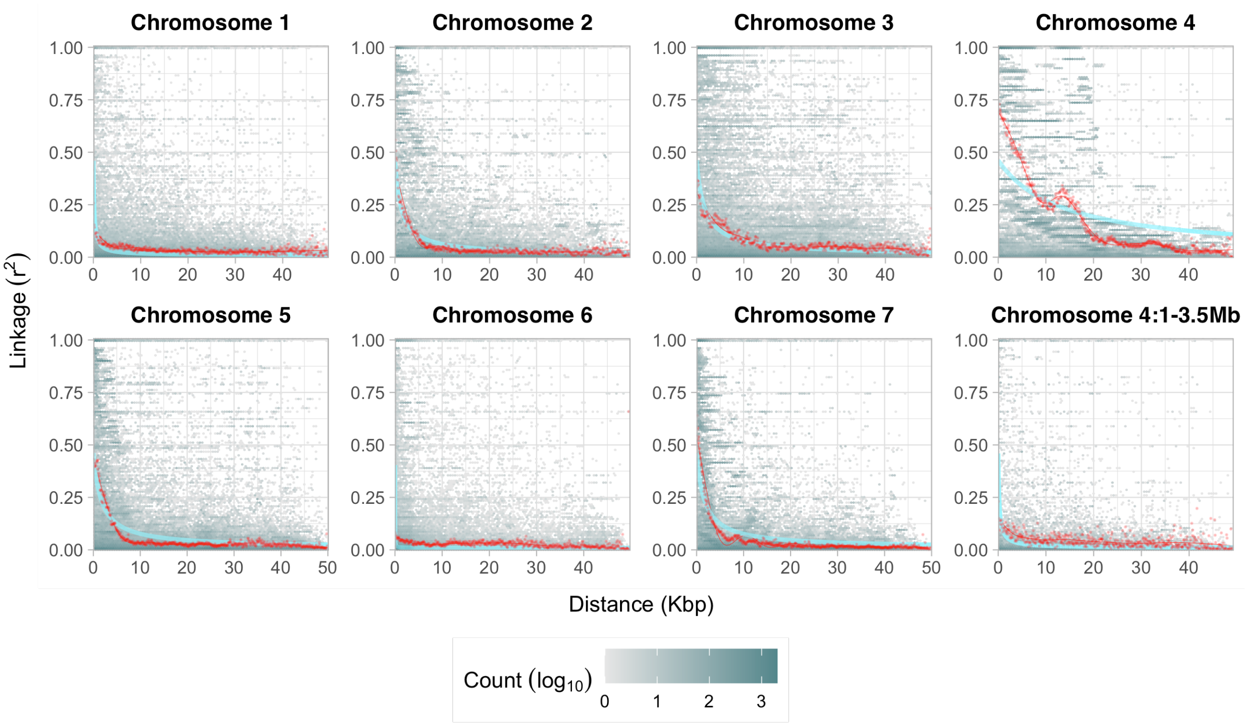

Supplementary Figure 3.

Linkage disequilibrium decay in the Wageningen collection of *P. anserina*. The r^2 statistic was estimated from within 30 windows (50 kb long) randomly sampled along each chromosome. Hexagonal bins are coloured according to the *log10* count of measurements for a given distance vs r^2 combination. The light blue curve corresponds to a nonlinear regression model following Remington et al.[4], while the red line is a generalized additive mode smoothing. Red points correspond to mean r^2 values of 1 kb distance bins.

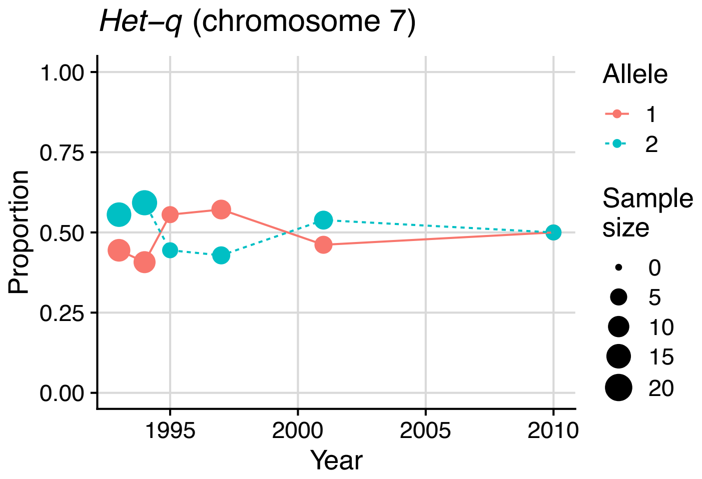

Supplementary Figure 4.

Changes of allele frequencies through time for *het-q* (data shown from years with more than five samples).

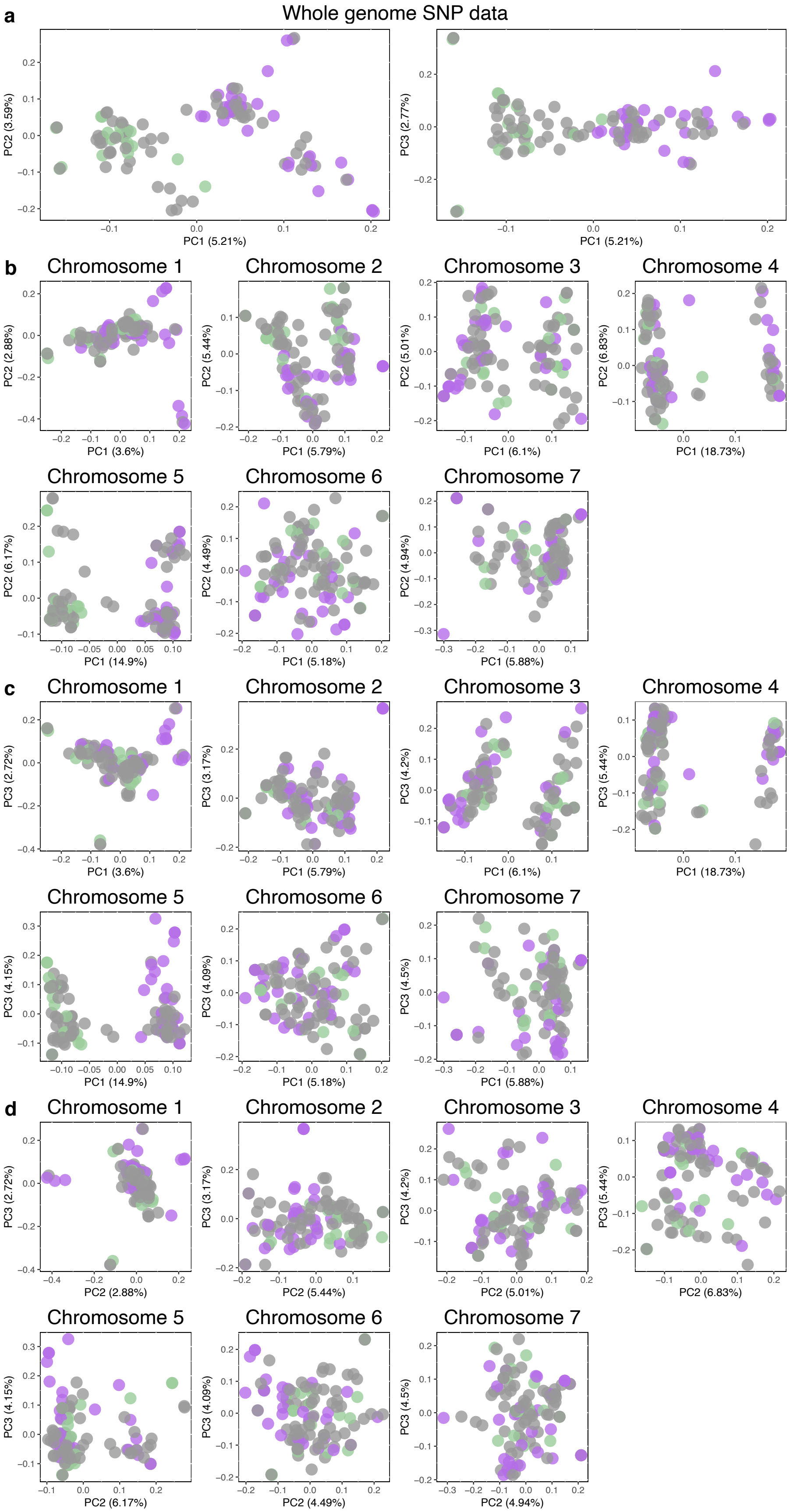

a
Whole genome SNP data
PC1 (5.21%)
PC2 (3.59%)
PC3 (2.77%)
b
Chromosome 1
Chromosome 2
Chromosome 3
Chromosome 4
Chromosome 5
Chromosome 6
Chromosome 7
PC1 (3.6%)
PC1 (5.79%)
PC1 (6.1%)
PC1 (18.73%)
PC1 (14.9%)
PC1 (5.18%)
PC1 (5.88%)
PC2 (2.88%)
PC2 (5.44%)
PC2 (5.01%)
PC2 (6.83%)
PC2 (6.17%)
PC2 (4.49%)
PC2 (4.94%)
c
PC3 (2.72%)
PC3 (3.17%)
PC3 (4.2%)
PC3 (5.44%)
PC3 (4.15%)
PC3 (4.09%)
PC3 (4.5%)
d

Supplementary Figure 5. (above)

Clustering analysis of SNP data for the Wageningen Collection of *P. anserina*. (**a**) A PCA of SNP data from all chromosomes return two groups that mirror the mating data. The PCA of SNP data per chromosome are also shown, comparing PC1 vs PC2 (**b**), PC1 vs PC3 (**c**), or PC2 vs PC3 (**d**). Samples with mating data are coloured green or lilac based on their PCoA grouping of mating success (**Fig. 2a**). Gray points have no mating success data.

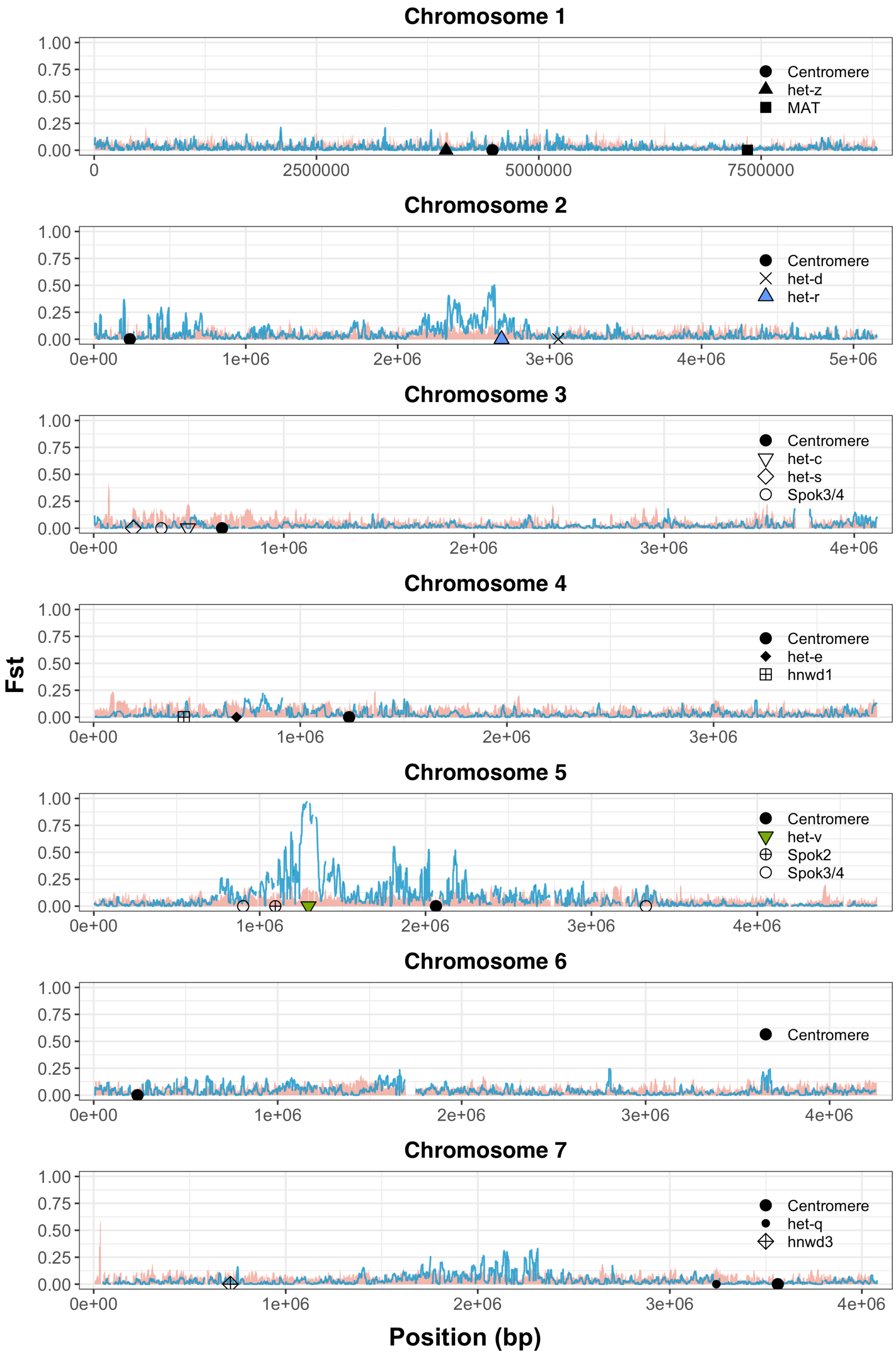

Supplementary Figure 6.

Genetic differentiation between the two mating groups. The F_{st} statistic was calculated in sliding windows (10kb-long with steps of 1kb) and plotted in dark blue. The light red areas represent the maximum F_{st} values reached by 1000 permutations of group membership. Marked loci include *het* genes, the centromere, the mating type locus (MAT), and meiotic drivers of the *Spok* family. Note that *Spok3* and/or *Spok4* can be found at different locations in the genome depending on the strain.

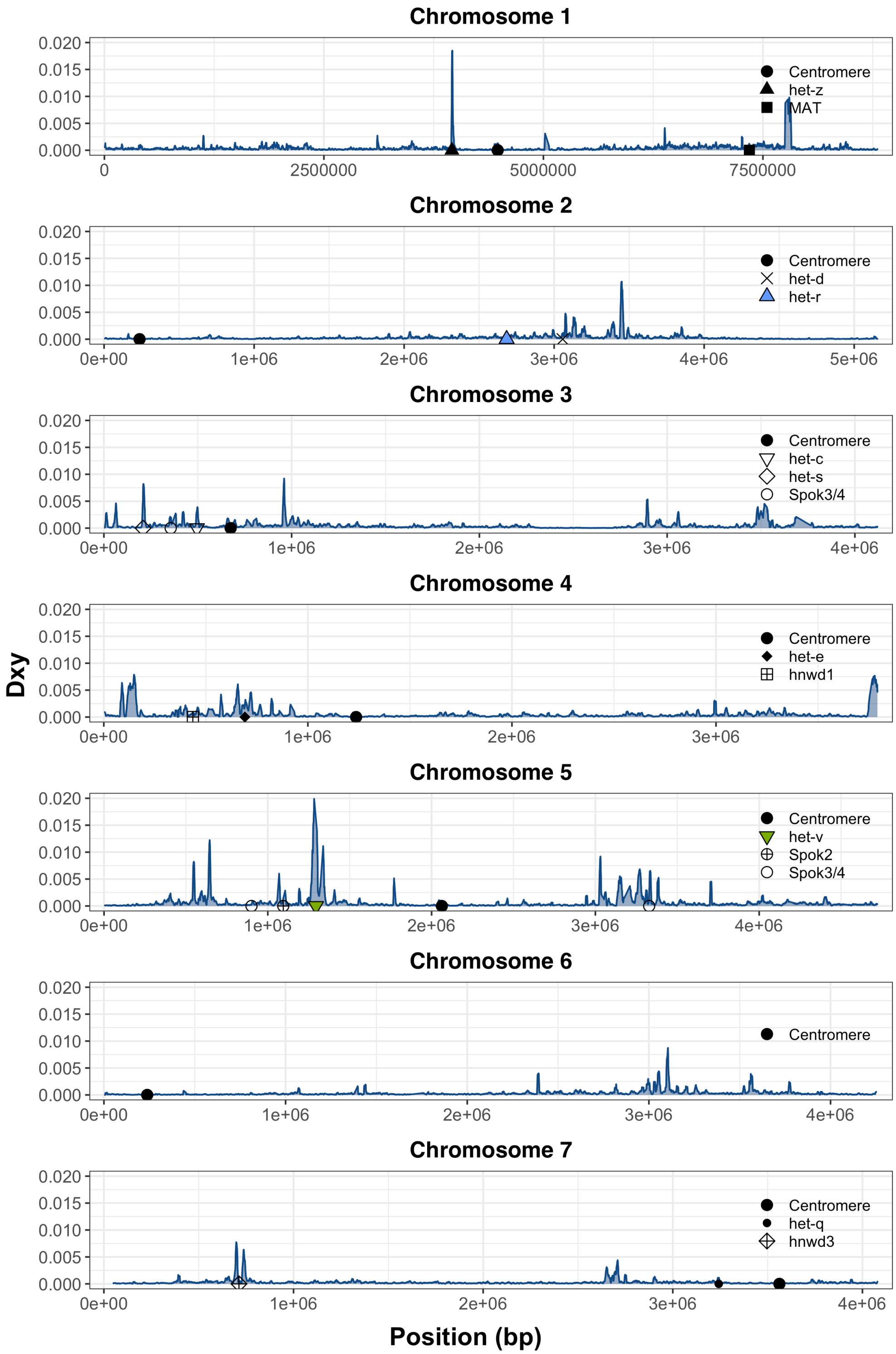

Supplementary Figure 7.

The pairwise nucleotide diversity between mating groups (D_{xy}) in sliding windows of 10 kb (1 kb steps). Relevant loci are marked, namely *het* genes, the centromere, and meiotic drivers of the *Spok* family. Note that *Spok3* and/or *Spok4* can be found at different locations in the genome depending on the strain.

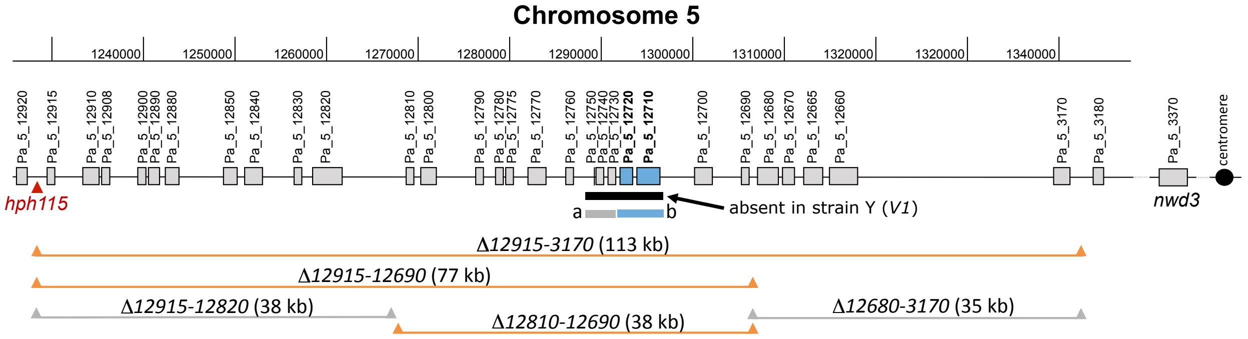

Supplementary Figure 8.

Deletion strategy to identify the genetic basis of *het-v*. Nested deletions between markers *hph115* and *nwd3* were made in the left arm of Chromosome 5, marked in orange if they lost the barrage formation in confrontations with a *V1* strain (i.e., became compatible), or in grey if they did not. Based on the absence of a number of genes in the genome of strain Y (of *V1* phenotype, marked in a black bar), two different fragments (*a* in grey and *b* in blue) were transformed into the *Δ12810-12690* strain as recipient to identify *het-v*.

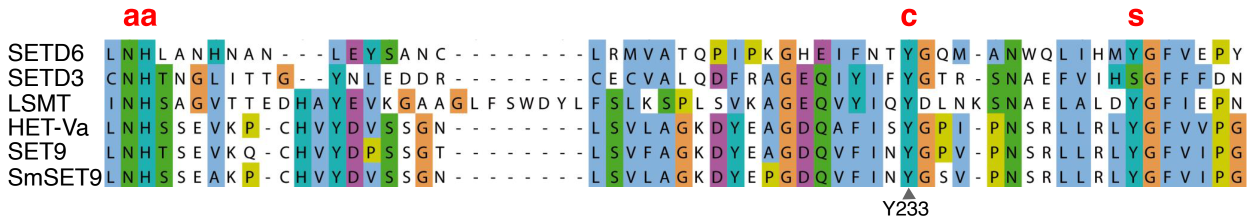

Supplementary Figure 9.

Alignment of HET-Va with *Neurospora crassa* SET9 and methyltransferases of known structure. The sequences correspond to the human SETD6, the human SETD3, the Rubisco LSMT methyltransferase from *Pisum sativum*, SET9 from *N. crassa*, and the SET9/HET-Va homolog from *Sordaria macrospora.* Positions highlighted are responsible for various functions in the proteins of known function (a=Adomet binding, s=substrate binding, c=catalysis). Mutated residue Y233 is marked with a triangle.

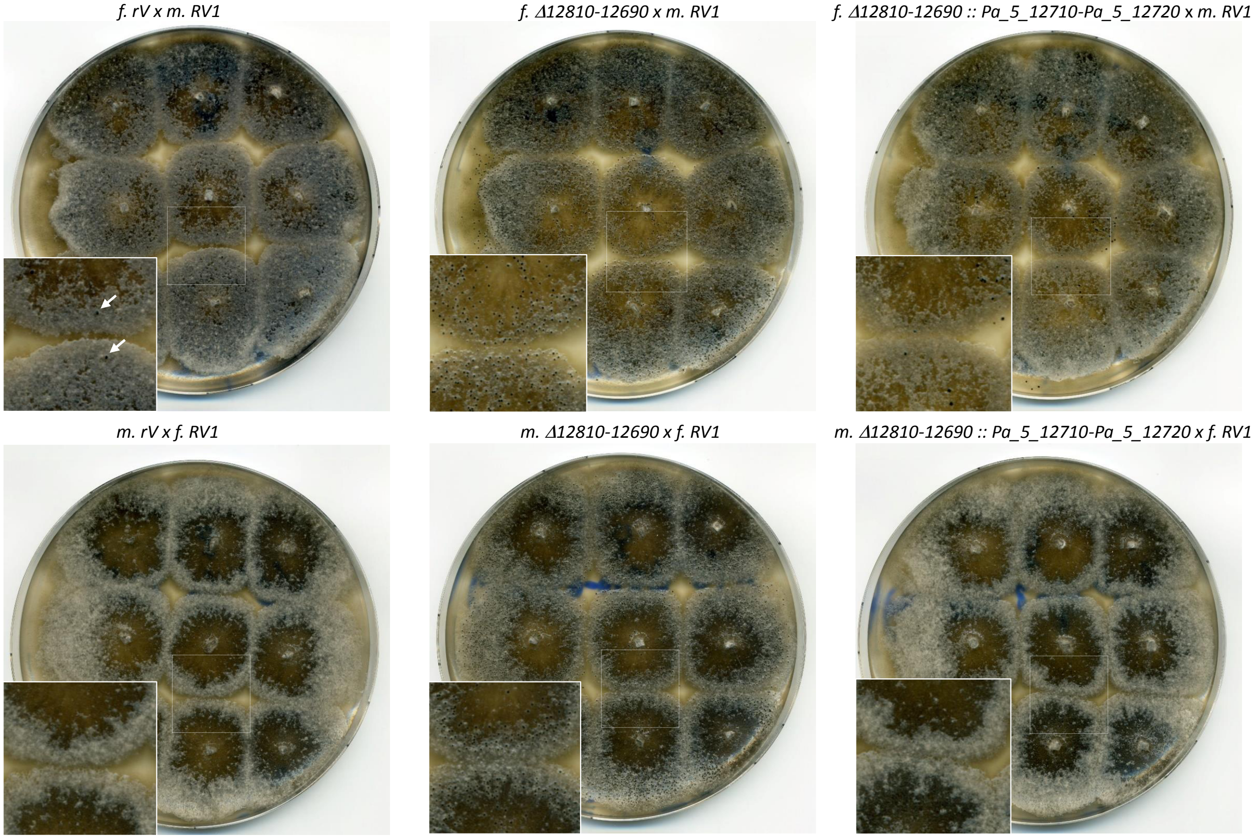

Supplementary Figure 10.

The region encompassing Pa_5_12710 (*het-Vb*) and Pa_5_12720 (*het-Va*) is responsible for low fertility of *rV* x *RV1* crosses. Crosses were set up between the given genotypes by fertilization (also known as spermatization). In the top row, the haploid *RV1* strain is used as male (*m.*) parent to fertilize a haploid female (*f.*) parent. In the bottom row, the *RV1* strain is used as female parent. The white arrows point to rare fruiting bodies. In both directions, the *Δ12810-12690* deletion restores full fertility. Reintroduction of the region encompassing the Pa_5_12710 and Pa_5_12720 genes in the deletion strain reduces fertility, indicating that this region is responsible for the sexual incompatibility. The reduction on fertility is stronger when the female parent is of *RV1* genotype.

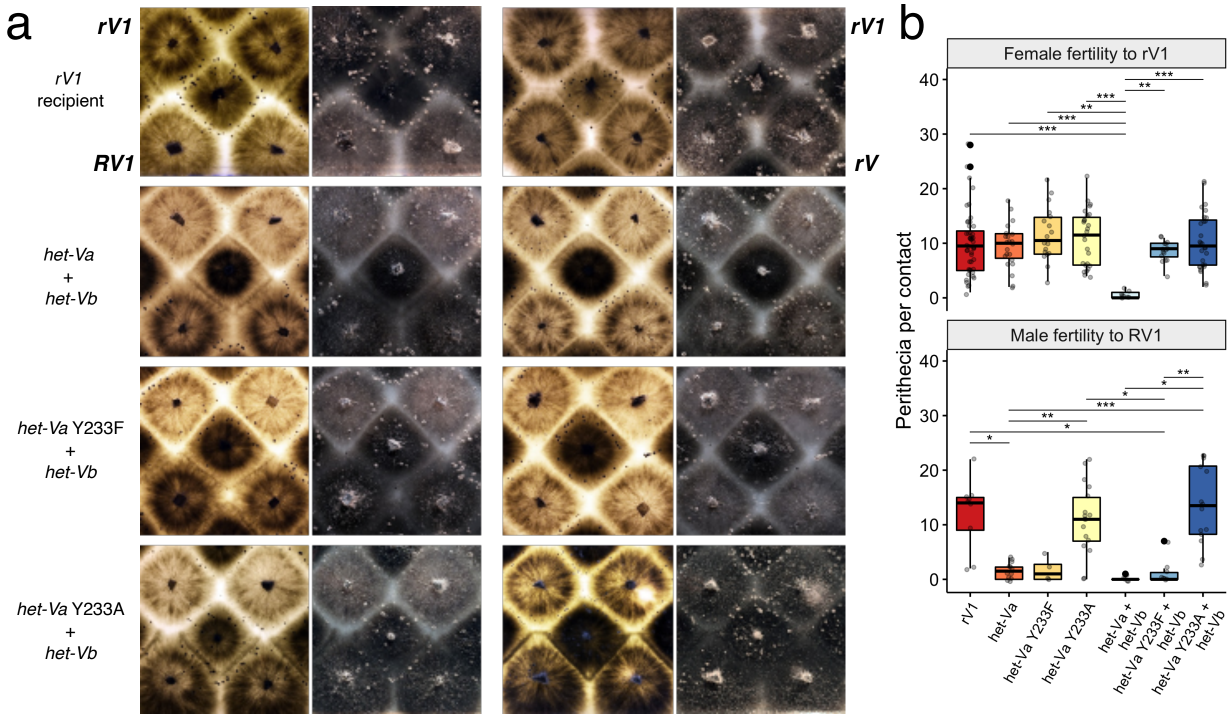

Supplementary Figure 11.

Fertility of transformants expressing mutant forms of the *het-Va* gene product. An *rV1* recipient strain was transformed with the indicated genes (wild-type and mutant *het-Va* together or without wild-type *het-Vb*). Transformants were confronted to *rV1*, *RV1* and *rV* tester strains and incubated in the light for a week until perithecia developed. (**a**) Photographs of the focal strain in the centre surrounded by tester strains (*rV1*, *RV1* or *rV*) are given. Each strain is shown illuminated from the bottom (left panel, light colour) or the top (right panel, dark colour) to facilitate visualization of the perithecia. In (**b**) female fertility in a confrontation with a *rV1* male (top) was quantified by counting the number of perithecia located on the focal strain on the half colony adjacent to the *rV1* strain. Male fertility in confrontation with a *RV1* female (bottom) was quantified by counting the number of perithecia located on the *RV1* strain in half colony adjacent to the focal strain. The differences between focal strains per condition (top or bottom) are significant (Kruskal-Wallis test, df = 6, $X^2_{female} = 40.862$, $p_{female} = 3.083e\text{-}07$, n = 165; $X^2_{male} = 26.383$, $p_{male} = 0.0001888$, n = 69). Horizontal lines inside boxplots represent the median and the lower and upper limits of the 25th and 75th percentiles, with whiskers as additional 1.5 x the interquartile range. Raw counts are shown as grey dots. Significance level is given by post-hoc two-sided pairwise Wilcoxon tests with Bonferroni correction: * = $p < 0.05$, ** = $p < 0.01$, *** = $p < 0.001$ (only significant comparisons are shown).

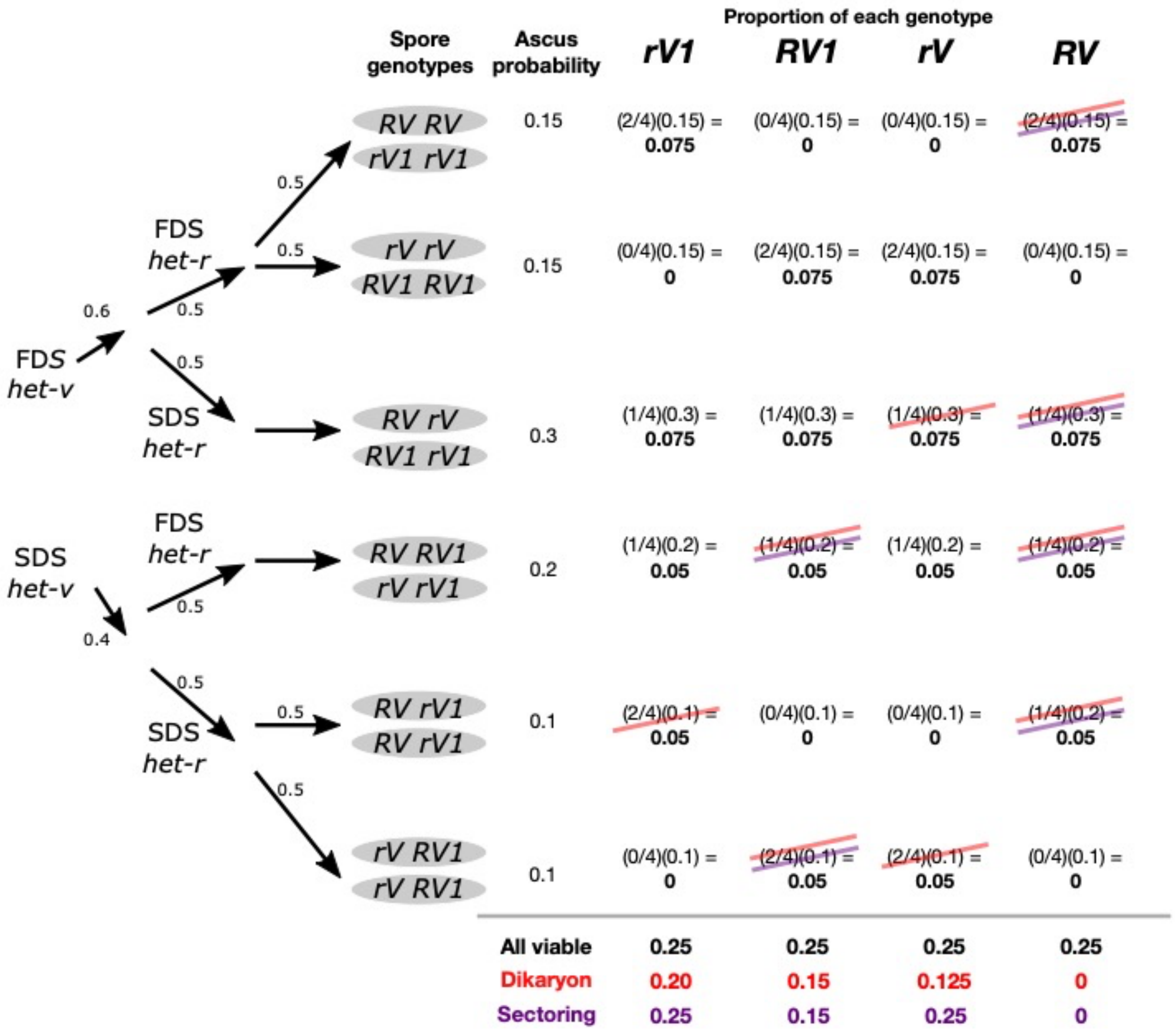

Supplementary Figure 12.

Patterns of segregation of *het-r* and *het-v*. In *P. anserina* the products of meiosis are duplicated by a mitosis event, and then rearranged in paired non-sister nuclei within two pairs of identical spores per ascus (only two shown for simplicity). A given locus might undergo first or second division segregation (FDS and SDS) during meiosis with a frequency dependent on the distance to the centromere. As *het-r* and *het-v* are on different chromosomes, their segregation is independent, which would result in equal proportions of the resulting spore genotypes if there was no incompatibility (0.25 each). As the *R* and *V* alleles are incompatible, a fraction of the spores dies upon germination, but the exact proportion is unknown. If a dikaryotic spore contains one *RV* nucleus, the incompatibility reaction might also kill the other nucleus during germination (red lines), in which case only 47.5% of all the nuclei produced by the cross survive. Alternatively, the other nucleus might escape by sectoring into a haploid mycelium while the *RV* counterpart dies (75% survival). As the *V* product is diffusible, it might still kill the sectoring *RV1* nuclei (purple lines). That would result in the survival of 65% of nuclei per cross.

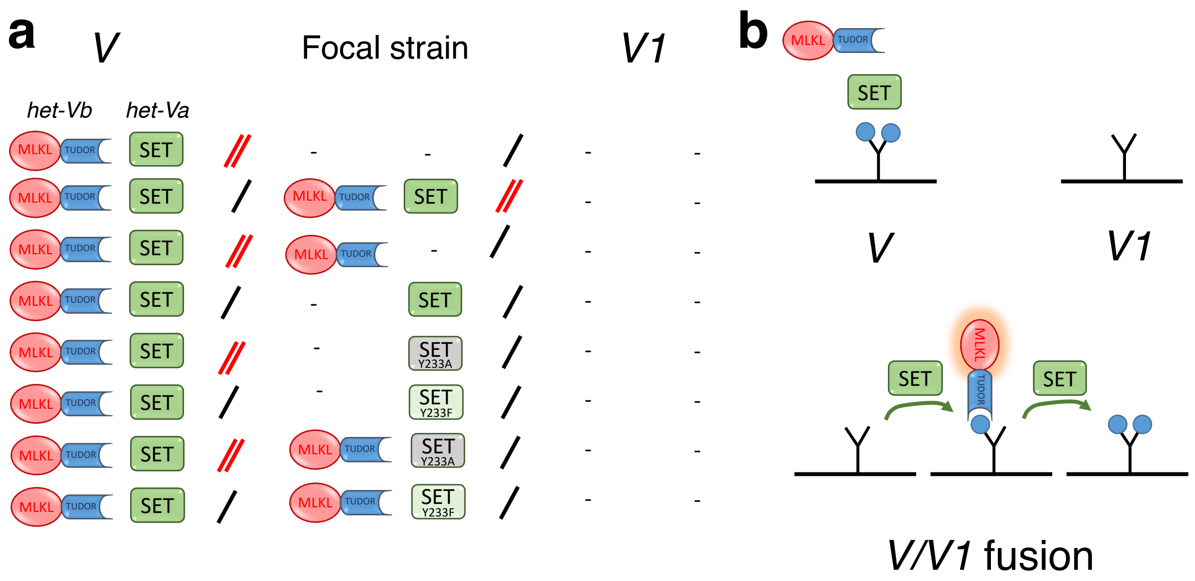

Supplementary Figure 13.
Tentative mechanistic model for allelic *V*/*V1* incompatibility. (**a**) The results of the expression of *het-Va* and *het-Vb* in a *V1* strain given in table S4 are depicted in a diagram form. The *het-Va* and *het-Vb* products present in the *V* and *V1* tester strains and the transformed *V1* recipient strain are represented with their relevant protein domains (MLKL, TUDOR and SET). The red double bar denotes a barrage reaction (incompatibility), the single black bar a compatible interaction. Joint expression of *het-Va* and *het-Vb* is required to confer the *V* phenotype (incompatibility to *V1*). Expression of *het-Vb* alone has the same phenotype as the absence of both genes (same phenotype as *V1* recipient). In contrast, expression of *het-Va* alone abolishes incompatibility to *V*. Note that the two mutants of *het-Va* (in grey and light green) have different phenotypes. While *het-Va* Y233A behaves as null mutant (with respect to allelic *V/V1* incompatibility), *het-Va* Y233F has a wild-type behaviour, in the sense that this allele abolishes incompatibility to *V*, as does wild-type *het-Va*. Yet, in contrast to wild-type, joined expression of *het-Va* Y233F with *het-Vb* does not confer incompatibility to *V1*. The phenotype conferred by *het-Va* Y233F is thus different both from wild-type and from *het-Va* Y233A. The activity of the *HET-Va* Y233F allele could be explained by a potential dominant negative effect on the methyltransferase activity of wild-type *het-Va* or by a residual activity of this more conservative change. (**b**) A possible mechanistic model for *V/V1* incompatibility based on the experimental results presented in (a) and on the predicted functions of the HET-Va and HET-Vb protein domains is given. In this model, HET-Va SET domain protein deposits a methylation mark on an unknown cellular target. This target is thus marked as "self" in *V* strains and not recognized by the HET-Vb *reader* and cell death inducing protein. In *V1* strains, both proteins are absent and the target is not marked. Upon fusion of *V* and *V1* strains, unmarked targets are detected as "non-self" by the HET-Vb *reader* which induces cell death. The fact that expression of HET-Vb alone in *V1* does not lead to incompatibility rules out a simple scenario where HET-Vb directly recognizes the unmarked target. Rather, presence of HET-Va is also required to bring about cell death. This could occur for instance if HET-Vb recognizes a methylation state formed transiently when naive (*V1*-type target) is exposed to HET-Va, as depicted in the graphic model.

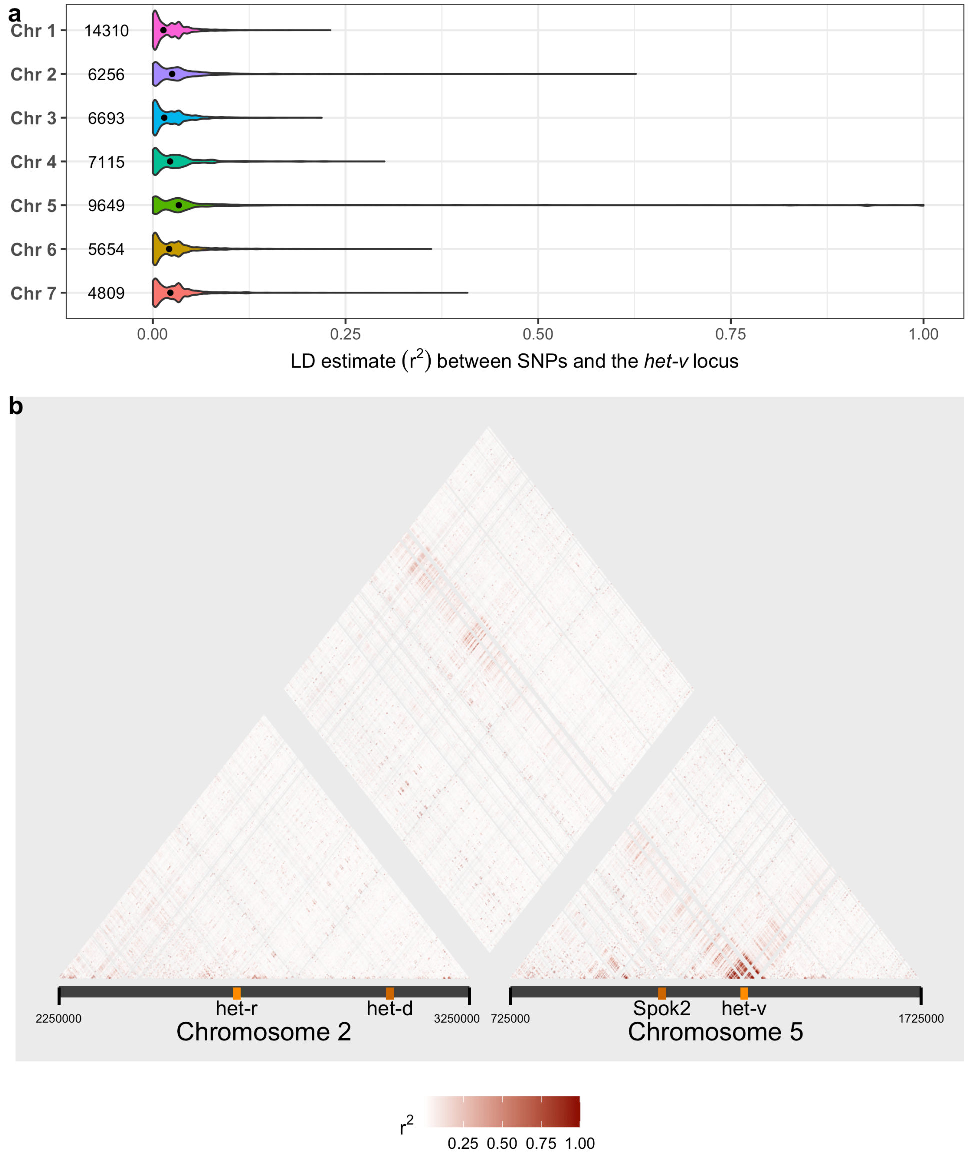

Supplementary Figure 14.

Linkage disequilibrium (LD) of *het-v* with other regions in the genome. **A**. Violin plot of LD values (r^2) between *het-v* and biallelic SNPs in all chromosomes. The median r^2 of each chromosome is marked with a black dot. The number of SNPs used per chromosome are indicated to the left of the distributions. **B.** A heatmap of intra- (triangles) and inter-chromosomal (diamond) LD in 1 Mb from chromosome 2 and 5 containing *het-r* and *het-v*, respectively. While most LD values are less than 0.2, there seems to be a strong association of SNPs surrounding *het-v*. There is also a modest intra-chromosomal association matching the location of the *het-v* linkage-block with the area surrounding *het-r*. The location of relevant *het* genes and the meiotic driver *Spok2* is given. Notice that areas with repeat content and indels, including *Spok2*, *het-v* and *het-r* themselves, were removed from the analysis during the SNP filtering.

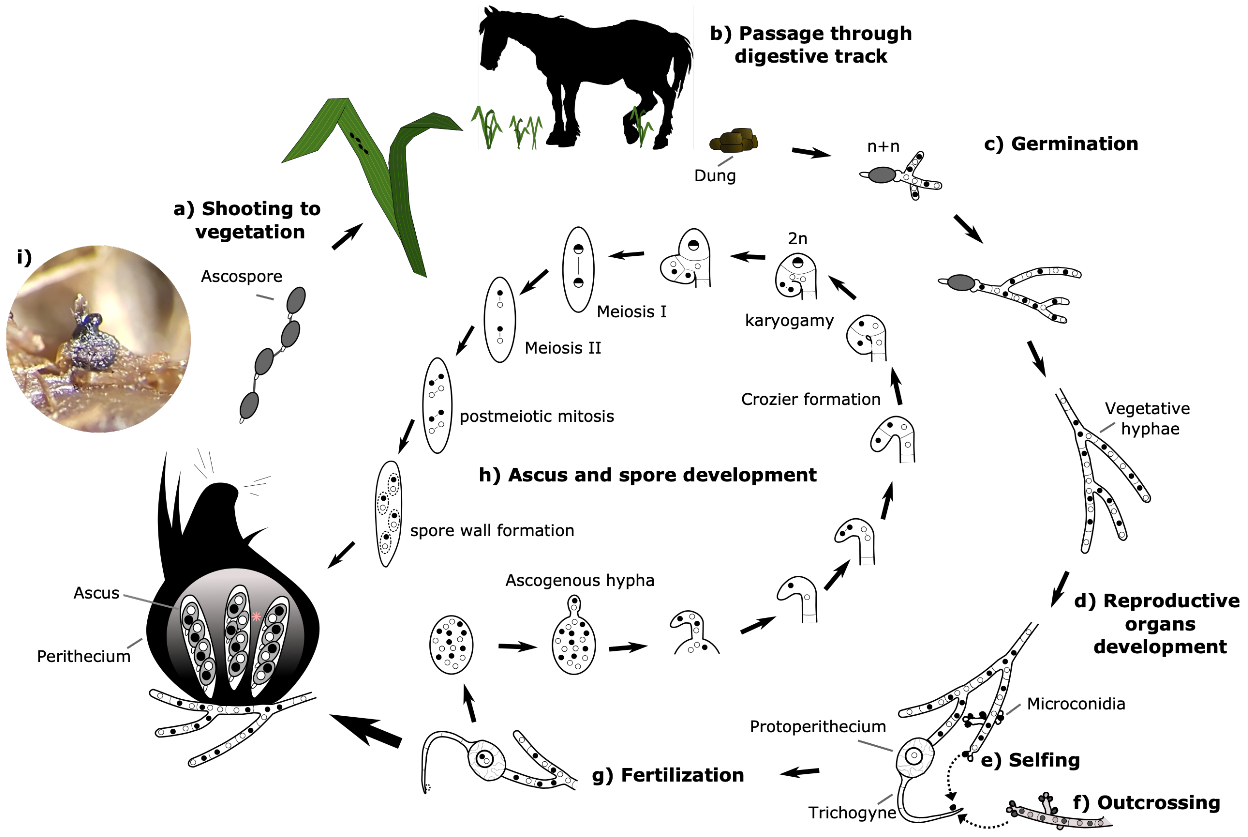

Supplementary Figure 15.

The life cycle of *Podospora anserina*. The fruiting bodies (perithecia) of *P. anserina* can be found in dung of herbivores. Once the sexual spores (ascospores) are fully developed, they are shot into the surrounding vegetation, where they get swallowed by for example a horse (**a**). The spores get activated by passing through the digestive track of the animal (**b**), and later deposited in the dung where they germinate (**c**). The mycelium continues growing somatically until starvation, when it differentiates reproductive organs (**d**). Like most ascomycetes, *P. anserina* is monoecious (hermaphroditic) with anisogamic gametes. It produces small gametes called microconidia (or spermatia), and large complex female structures named protoperithecia. The protoperithecium has a large projection termed trichogyne, which reacts to a compatible microconidum that might come from the same individual (**e**; selfing) or from a different one (**f**; outcrossing). The nucleus of the microconidium travels through the trichogyne to the protoperithecium's nucleus. After fertilization (**g**), an ascogenous hypha differentiates into a sac-like structure or ascus (**h**). During this process, the two haploid nuclei undergo karyogamy reaching a diploid state (2n). The cell immediately goes into meiosis I, II, and finally a postmitotic mitosis. The spore wall then forms around two of the haploid nuclei, producing dikaryotic spores (n+n). Occasionally haploid, self-incompatible spores are produced (pink asterisk). The picture of a perithecium in (**i**) was taken from horse dung in Wageningen in 2017 by S. L. Ament-Velásquez. Diagram based on refs. [5–7].

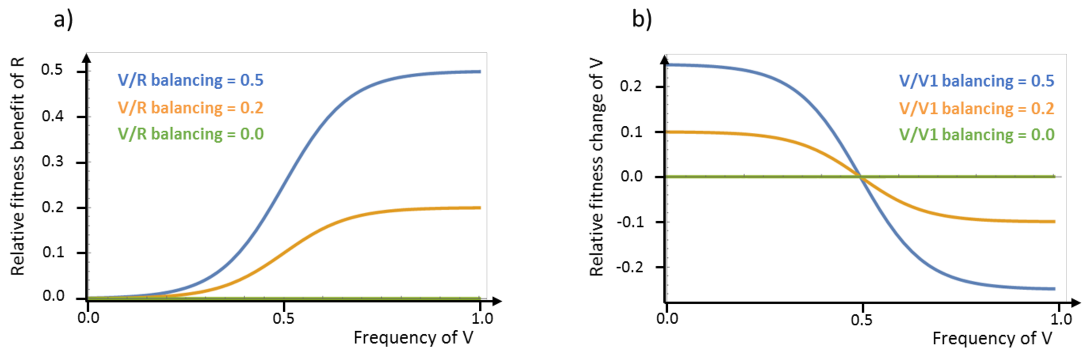

Supplementary Figure 16. Fitness function of balancing selection in the *V/R* and *V/V1* interactions. (**a**) Relative fitness benefit of *R* over *r* as a function of the frequency of *V* in the population (or *V* over *V1* as a function of the frequency of *R*), and the intensity of the *V*/*R* balancing selection parameter (represented with different colours). (**b**) Change in relative fitness of *V* as a function of its own frequency in the population, and the intensity of the *V*/*V1* balancing selection parameter.

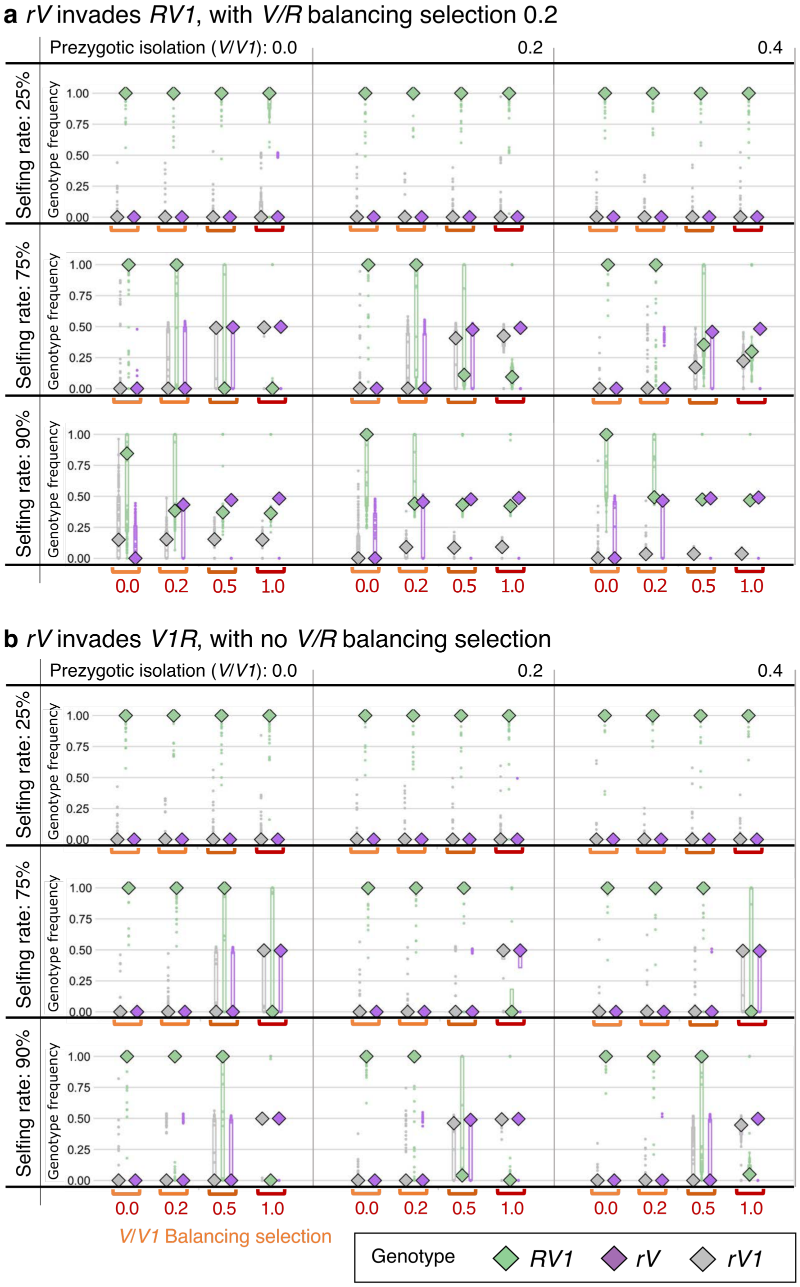

Supplementary Figure 17.

Summary of individual-base simulations of *rV* invading an *RV1* population with intensity of *V*/*R* balancing selection fixed to (**A**) 0.2 or (**B**) 0. For each parameter combination, the distribution of genotype frequencies of 100 replicated simulations is given. Each simulation is represented by a dot, and their distribution by a boxplot, which shows the median as a diamond, the 25th to 75th percentiles as the box bounds, and 1.5x interquartile ranges as whiskers. If dots and boxplots are not visible, the distribution is concentrated behind the diamond of the median.

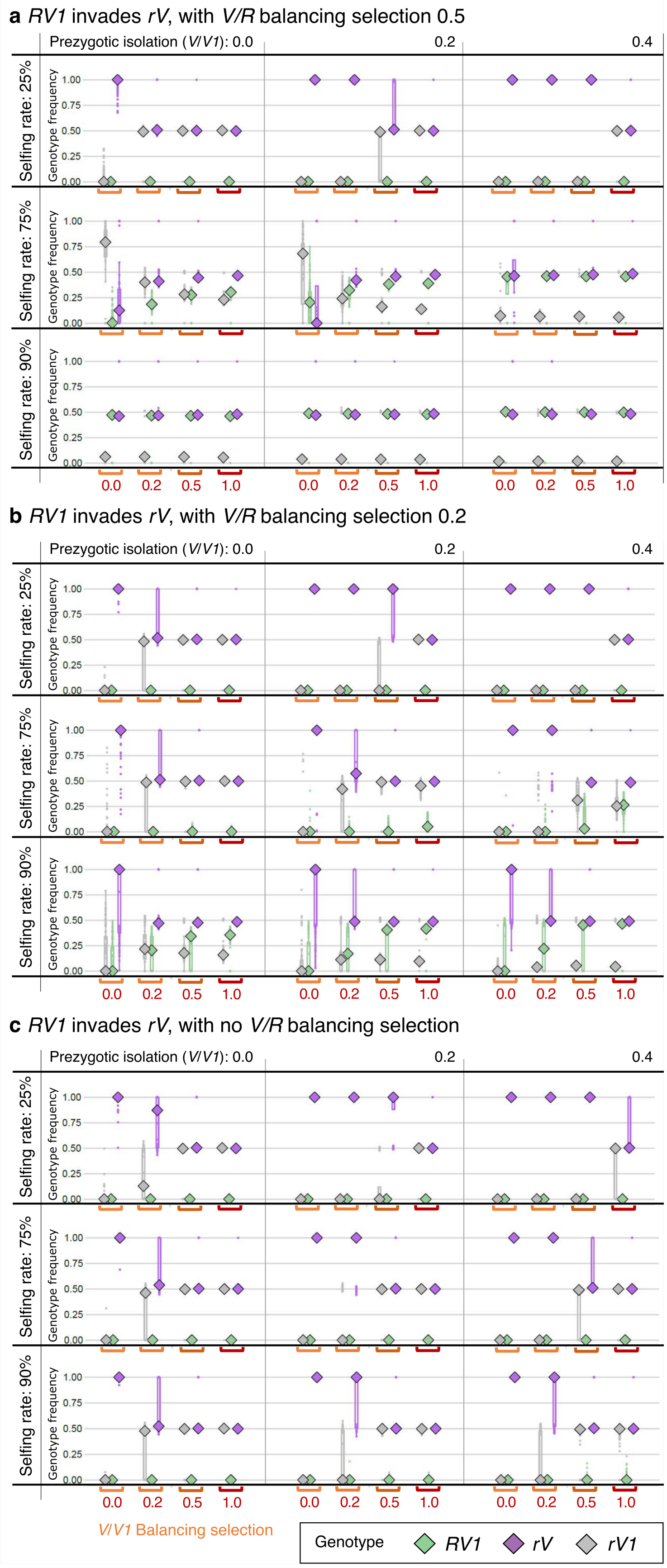

a RV1 invades rV, with V/R balancing selection 0.5
b RV1 invades rV, with V/R balancing selection 0.2
c RV1 invades rV, with no V/R balancing selection
Prezygotic isolation (V/V1): 0.0
0.2
0.4
Selfing rate: 25%
Selfing rate: 75%
Selfing rate: 90%
Genotype frequency
1.00
0.75
0.50
0.25
0.00
0.0
0.2
0.5
1.0
V/V1 Balancing selection
Genotype
RV1
rV
rV1

Supplementary Figure 18. (above)

Summary of individual-base simulations of *RV1* invading an *rV* population with intensity of *V*/*R* balancing selection fixed to (**A**) 0.5, (**B**) 0.2 or (**C**) 0. For each parameter combination, the distribution of genotype frequencies of 100 replicated simulations is given. Each simulation is represented by a dot, and their distribution by a boxplot, which shows the median as a diamond, the 25th to 75th percentiles as the box bounds, and 1.5x interquartile ranges as whiskers. If dots and boxplots are not visible, the distribution is concentrated behind the diamond of the median.

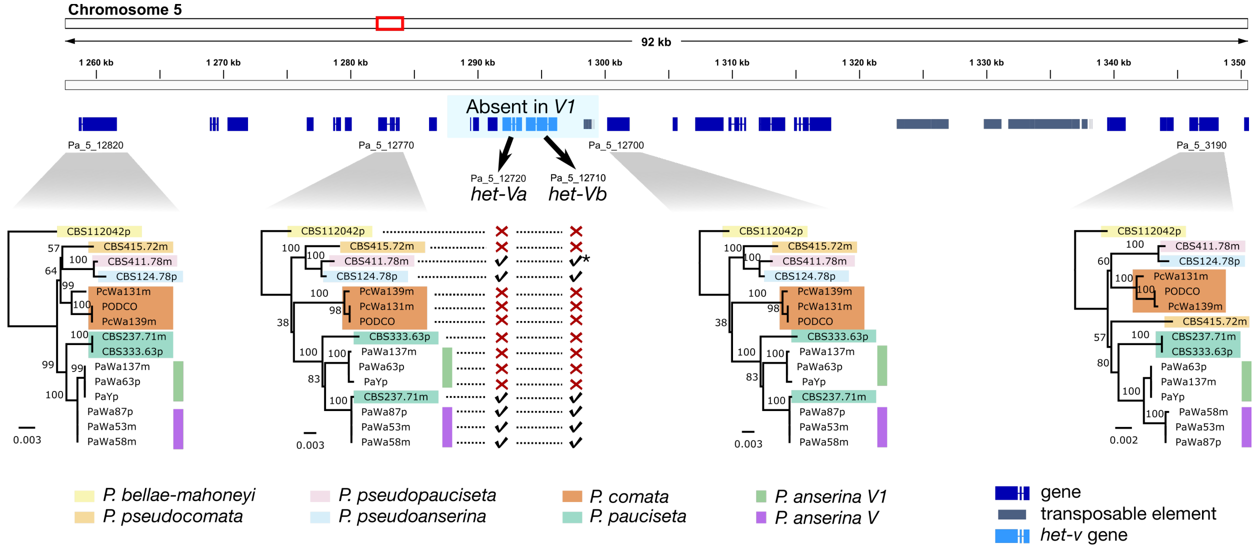

Supplementary Figure 19. Phylogenetic relationships of genes flanking the *het-v* locus. The presence and absence of the *het-Va* and *het-Vb* genes are marked for all strains. Outside *P. anserina*, *het-v* is only found in *P. pseudoanserina* (CBS 124.78) and *P. pseudopauciseta* (CBS 411.78), although *het-Vb* is truncated in the latter (marked with an asterisk). The locus equivalent to the *V1* allele (absence of *het-Va* and *het-Vb*) was found populated by different transposons or simply absent in other strains. While genealogies of genes far from the *het-v* locus recover *P. pauciseta* as monophyletic (i.e., CBS 237.71 and CBS 333.63 are sisters), in the genealogies of genes next to *het-v* the strain CBS 237.71 nests within the clade of *P. anserina* strains carrying the *V* allele. Rather than trans-species polymorphism, the topologies suggest introgression between *P. anserina* and *P. pauciseta*, as the strain CBS 333.63 maintains the expected position in the species tree (i.e. it does not cluster with the *V1* allele of *P. anserina*). In the absence of population data of all species, it remains unclear if the presence of *het-v* is ancestral to the species complex. However, the RI groups in *P. anserina* cluster together for all genes, confirming that the divergence beyond the *het-v* locus happened after *P. anserina* diverged from other species. Branch support (bootstraps) is indicated above the tree branches. Branch lengths are proportional to the scale bar (nucleotide substitution per site). Rooting was done arbitrarily with CBS112942.

Supplementary Table 1.

Sampling information and genotyping of the *P. anserina* strains used in this study. The columns cover the mating type of the monokaryon sequenced (MAT), the year of collection, the locality, the substrate source (herbivore), the allele of known *het* genes, whether they had mating data from van der Gaag[8] available, and whether the whole genome sequencing was done in this study or elsewhere. The phenotype of *het-r* ("Het-r_phen") was determined based on the number of WD40 repeats and their sequence when available ("Verification_het-r"). The alleles of *het-c* (based on the DNA sequence) where further categorized into the phenotypic classes as defined in ref. [9] in the column "Het-c_phen". In the columns of *Spok* genes, 1 means presence and 0 means absence.

Supplementary Table 2.
Sampling details of the strains isolated in 2017.

Supplementary Table 3.
Matrix of mating success scores between strains of the Wageningen Collection, based on the results of van der Gaag[8].

Supplementary Table 4.
Vegetative incompatibility phenotype of an *rV Δ12810-12690* recipient strain (confronted to *rV*, *RV1*, and *rV1*) after being transformed with wild-type and mutant *het-Vb* and *het-Va*

DNA	*rV*	*RV1*	*rV1*	phenotype
vector	//	/	/	*V1*
fragment b (*het-Vb*, *het-Va*)	/	//	//	*V*
het-Vb and *het-Va*	/	//	//	*V*
het-Vb	//	/	/	*V1*
het-Va	/	//	/	novel
het-Va Y233A	//	//	/	novel
het-Va Y233F	/	//	/	novel
het-Vb and *het-Va* Y233A	//	//	/	novel
het-Vb and *het-Va* Y233F	/	//	/	novel

// denotes an incompatible contact (barrage); / a compatible contact

Supplementary Table 5.
Raw counts of perithecia formed in confrontations of *het-Va* point mutations and control crosses.

Supplementary Table 6.

Primers used for the amplification of the WD40 domain in *het-r*[10]. Location of primers corresponds to their position in the *het-r* locus (compared to start codon) from *P. anserina* genome of the S strain.

Primer	Sequence	Location
WD_A_F	GCA CCG GTT GGC AGT CTG G	Upstream from WD domain (+2388 to +2406)
WD_B_R	CCA GGC CCT TCT CGT GTT AGG	Terminator (+3150 to +3170)
WD_F_R	GAA CTC CTA TAG CCT TCG AG	Downstream from WD domain (+2871 to +2890)
WD_G_F	GAA TGC ATG CCT TCA GAC G	Upstream from WD domain (+2474 to +2492)

Supplementary Table 7.

Distribution of the *het-Va* (Pa_5_12720) and *het-Vb* (Pa_5_12710) genes in the genomes of various *Neurospora* species.

References

1. Haller, B. C. & Messer, P. W. SLiM 3: Forward Genetic Simulations Beyond the Wright-Fisher Model. *Molecular Biology and Evolution* **36**, 632–637 (2019).
2. Bernet, J. Les systèmes d'incompatibilité chez le *Podospora anserina*. *Comptes rendus hebdomadaires des séances de l'Académie des sciences, Série D* **265**, 1330–1333 (1967).
3. Dalstra, H. J. P., Swart, K., Debets, A. J. M., Saupe, S. J. & Hoekstra, R. F. Sexual transmission of the [Het-s] prion leads to meiotic drive in *Podospora anserina*. *Proc Natl Acad Sci U S A* **100**, 6616–6621 (2003).
4. Remington, D. L. *et al.* Structure of linkage disequilibrium and phenotypic associations in the maize genome. *Proceedings of the National Academy of Sciences* **98**, 11479–11484 (2001).
5. Saupe, S. J. A short history of small s: a prion of the fungus *Podospora anserina*. *Prion* **1**, 110–115 (2007).
6. Pinan-Lucarré, B., Paoletti, M. & Clavé, C. Cell death by incompatibility in the fungus *Podospora*. *Seminars in Cancer Biology* **17**, 101–111 (2007).
7. Ames, L. M. Hermaphroditism Involving Self-Sterility and Cross-Fertility in the Ascomycete *Pleurage anserina*. *Mycologia* **26**, 392–414 (1934).
8. van der Gaag, M. *Genomic conflicts in* Podospora anserina. PhD thesis, Wageningen Universiteit (2005).
9. Bastiaans, E. *et al.* Natural variation of heterokaryon incompatibility gene het-c in Podospora anserina reveals diversifying selection. *Molecular Biology and Evolution* **31**, 962–974 (2014).
10. Chevanne, D. *et al.* Identification of the het-r vegetative incompatibility gene of Podospora anserina as a member of the fast evolving HNWD gene family. *Current Genetics* **55**, 93–102 (2009).
