# Supplementary material for: Allorecognition genes drive reproductive isolation in Podospora anserina
Source: Nat Ecol Evol. 2022 May 12;6(7):910–23. doi: 10.1038/s41559-022-01734-x (PMC9262711; doi:10.1038/s41559-022-01734-x)
Supplement: Supplementary file 2 — Reporting Summary. [file 41559_2022_1734_MOESM2_ESM.pdf]

nature portfolio

Corresponding author(s): Sandra Lorena Ament-Velásquez, Hanna Johannesson

Last updated by author(s): Aug 17, 2021

Reporting Summary

Nature Portfolio wishes to improve the reproducibility of the work that we publish. This form provides structure for consistency and transparency in reporting. For further information on Nature Portfolio policies, see our Editorial Policies and the Editorial Policy Checklist.

Statistics

For all statistical analyses, confirm that the following items are present in the figure legend, table legend, main text, or Methods section.

n/a	Confirmed	
☐	☒	The exact sample size (*n*) for each experimental group/condition, given as a discrete number and unit of measurement
☒	☐	A statement on whether measurements were taken from distinct samples or whether the same sample was measured repeatedly
☐	☒	The statistical test(s) used AND whether they are one- or two-sided<br>*Only common tests should be described solely by name; describe more complex techniques in the Methods section.*
☒	☐	A description of all covariates tested
☒	☐	A description of any assumptions or corrections, such as tests of normality and adjustment for multiple comparisons
☐	☒	A full description of the statistical parameters including central tendency (e.g. means) or other basic estimates (e.g. regression coefficient) AND variation (e.g. standard deviation) or associated estimates of uncertainty (e.g. confidence intervals)
☐	☒	For null hypothesis testing, the test statistic (e.g. *F*, *t*, *r*) with confidence intervals, effect sizes, degrees of freedom and *P* value noted<br>*Give P values as exact values whenever suitable.*
☒	☐	For Bayesian analysis, information on the choice of priors and Markov chain Monte Carlo settings
☒	☐	For hierarchical and complex designs, identification of the appropriate level for tests and full reporting of outcomes
☒	☐	Estimates of effect sizes (e.g. Cohen's *d*, Pearson's *r*), indicating how they were calculated

Our web collection on statistics for biologists contains articles on many of the points above.

Software and code

Policy information about availability of computer code

Data collection	Most of the data used was generated in this study, with the exception of the raw mating success data (as explained in the Main text and Methods) and the genome sequence of a few strains from the same population published previously (accession numbers available in the Supplementary Table 1). The new strains collected in 2016 and 2017 were deposited in the collection of Wageningen University & Research.
Data analysis	All code (in python, Snakemake and R) used for the bioinformatic analyses is available at the GitHub repository https://github.com/johannessonlab/HetVPaper

For manuscripts utilizing custom algorithms or software that are central to the research but not yet described in published literature, software must be made available to editors and reviewers. We strongly encourage code deposition in a community repository (e.g. GitHub). See the Nature Portfolio guidelines for submitting code & software for further information.

Data

Policy information about availability of data

All manuscripts must include a data availability statement. This statement should provide the following information, where applicable:

- Accession codes, unique identifiers, or web links for publicly available datasets
- A description of any restrictions on data availability
- For clinical datasets or third party data, please ensure that the statement adheres to our policy

Whole genome sequencing was deposited in NCBI under BioProject PRJNA743020. All other data is available as supplementary tables. The associated code is available in the GitHub repository https://github.com/johannessonlab/HetVPaper.

Field-specific reporting

Please select the one below that is the best fit for your research. If you are not sure, read the appropriate sections before making your selection.

☐ Life sciences ☐ Behavioural & social sciences ☒ Ecological, evolutionary & environmental sciences

For a reference copy of the document with all sections, see nature.com/documents/nr-reporting-summary-flat.pdf

Ecological, evolutionary & environmental sciences study design

All studies must disclose on these points even when the disclosure is negative.

Study description	In this study we used a Dutch collection of 106 strains from the fungus Podospora anserina to assess the connection between vegetative and sexual incompatibility and the effects on speciation. Using whole genome Illumina sequencing, we characterized the genetic diversity of this population and detected signatures of balancing selection for known vegetative incompatibility loci (het genes). Using previously published mating success data for the same population, we identified two reproductively isolated groups defined by the antagonistic interaction of two het genes: het-r and het-v. While het-r was already known, here we characterize het-v through positional and complementation cloning, as well as site-directed mutagenesis. We used substrate information of the collection, plus additional sampling of 68 strains to determined that the two reproductively isolated groups co-occur in their known substrate. Finally, we used individual-based simulations to gain insights into the evolution of this system.
Research sample	The Wageningen Collection consist of strains isolated around the city of Wageningen, The Netherlands, as described in Materials and Methods. Sequenced strains were collected between 1991 to 2016. Additional strains were sampled in 2017 but not sequenced. This fungus has a "pseudo-homothallic" reproductive system, meaning that the mycelium contains two types of haploid nuclei, one of each mating type, which allows for self-fertilization. It occasionally produces haploid ascospores, which were used for all the analyses. Here, a strain correspond to a single ascospore extracted from a fruiting body present in herbivore dung, as well as all its descendants derived by selfing. The Wageningen collection has been studied extensively for the evolution of meiotic drive, het genes, and senescence. This includes the original raw data of mating success found in the PhD thesis of Marjin van der Gaag, and which we re-analyzed here as described in Materials and Methods.
Sampling strategy	We attempted to include all the strains of the Wageningen Collection, but the spores of a few strains failed to germinate or had failed sequencing and were not included in the project. We also attempted to include available strains from other parts of the world (France, Switzerland, Canada, and Argentina), but very few strains of this and related species are available elsewhere.
Data collection	Sequencing, sampling, phenotyping and genotyping is described in the Materials and Methods section. Sampling of new strains in 2016 and 2017 was done by the authors SLAV, SDG, EB, and AJMD. Coding of mating success data was done by AAV. Phenotyping to assign strains from 2017 to the reproductively isolated groups was done by AAV and SLAV. Cloning, mutagenesis, and genotyping of het-v was done by AGF and CC. Preparation of cultures for sequencing was done by EB. All simulation analyses were done by IMA.
Timing and spatial scale	Samples were collected between 1991 and 2017 in association with several PhD projects from Wageningen University & Research and Uppsala University, as well as the course of Advanced Genetics imparted by AJMD. Isolating new strains is a laborious and specialized effort that is strongly subject to chance (e.g. mass mortality of the local rabbit population in some years).
Data exclusions	No data was excluded. Whenever the analyses included strains other than those sampled in Wageningen, this is specified in the Materials and Methods or in the Main text.
Reproducibility	The main bioinformatic analyses were done using the workflow manager Snakemake available in the GitHub repository of the paper. Otherwise, they can be reproduced from stand-alone R scripts also in the repository. The procedure to locate and characterize het-v, including failed attempts, are described in the Materials and Methods.
Randomization	All Wageningen samples were included in all analyses, as the objective was to characterize the population. The samples are only randomly assigned to groups to calculate the distribution of Fst values expected by chance.
Blinding	Blinding was not necessary as all the fungal strains are indistinguishable and there is no phenotypic trait associated to the reproductively isolated groups (other than the vegetative and sexual incompatibility itself)

Did the study involve field work? ☒ Yes ☐ No

Field work, collection and transport

Field conditions	Field work was done in grassland-like areas around Wageningen, the Netherlands, and strain isolation was done as described in Materials and Methods. Only the source herbivore for the dung was recorded.
Location	Wageningen, the Netherlands. The coordinates for the 2017 samples are provided in the Supplementary Table 2.
Access & import/export	As both Sweden and the Netherlands are parties of the Nagoya protocol, but have no Access and Benefit-Sharing legislation, a declaration of due diligence was not required.
Disturbance	The samples taken (pieces of dung from horses, cows, sheep and rabbit) were small and found mostly in agricultural areas or near roads.

Reporting for specific materials, systems and methods

We require information from authors about some types of materials, experimental systems and methods used in many studies. Here, indicate whether each material, system or method listed is relevant to your study. If you are not sure if a list item applies to your research, read the appropriate section before selecting a response.

Materials & experimental systems

n/a	Involved in the study
☒	☐ Antibodies
☒	☐ Eukaryotic cell lines
☒	☐ Palaeontology and archaeology
☐	☒ Animals and other organisms
☒	☐ Human research participants
☒	☐ Clinical data
☒	☐ Dual use research of concern

Methods

n/a	Involved in the study
☒	☐ ChIP-seq
☒	☐ Flow cytometry
☒	☐ MRI-based neuroimaging

Animals and other organisms

Policy information about studies involving animals; ARRIVE guidelines recommended for reporting animal research

Laboratory animals	The data associated to all Podospora strains used in this study is available in Supplementary tables 1 and 2.
Wild animals	The study involved fungal strains isolated from the wild as described above and in the Materials and Methods.
Field-collected samples	The maintenance and storage of the fungal strains is described in Materials and Methods.
Ethics oversight	For experimental work with Podospora anserina, no ethical approval or guidance was required.

Note that full information on the approval of the study protocol must also be provided in the manuscript.
